# Healthcare Professionals’ Views of the Integrated County Healthcare Consortium in Zhejiang, China

**DOI:** 10.5334/ijic.5690

**Published:** 2022-06-22

**Authors:** Qisheng Gao, Yao Ma, Pinghua Zhu, Dingwan Chen

**Affiliations:** 1 School of Public Health, Hangzhou Medical College, Hangzhou, 311399, CN; 2 School of Humanities and Social Sciences, Guangxi Medical University, Nanning 530021, CN

**Keywords:** healthcare professional, integrated county healthcare consortium, improvement measures, satisfaction

## Abstract

**Introduction:**

The integrated county healthcare consortium (ICHC) is becoming an important measure to improve the capacity of primary-level medical services and to achieve grading diagnosis and treatment system in China. However, it is not clear whether health professionals are satisfied with this reform and what are the problems with it. This study aimed to understand the satisfaction of healthcare professionals to the ICHC in Zhejiang Province, China, and analyze the problems and improvement measures of the ICHC.

**Methods:**

A cross-sectional study was carried out in the 11 pilot counties (cities and districts) implementing the construction of the ICHC in Zhejiang in November 2019. Healthcare professionals from the leading county-level hospital, three township health centers (THCs) or community health centers (CHCs) in each ICHC were invited to participate in this survey.

**Results:**

A total of 3531 healthcare professionals were included, 85.92% of the participants agreed that the integration of the Centers for Disease Control and Prevention (CDC) and other professional public health institutions into the construction of ICHC could actively promote basic public health work. The most severe problem was the lack of financial guarantee fund input (severity score: 2.92 ± 1.76). The most crucial measure to promote the construction of the ICHC was to increase government financial input and improve the security mechanism (importance score: 4.81 ± 0.47). The satisfaction of the healthcare professionals to the ICHC was 89.41%. The satisfaction of healthcare professionals from county-level hospitals was 2.37 (95% CI: 1.760–3.238) times higher than that of healthcare professionals from the township health centers (THCs) or community health centers (CHCs). The satisfaction of health professionals with a college degree or below was 3.215 (95% *CI*: 1.413–6.786) times higher than that of health professionals with a master’s degree or above.

**Conclusions:**

Zhejiang Province has taken adequate measures to promote the construction of the ICHC. However, there are still some problems. Appropriate and effective policies should be implemented to enhance policy coordination and promote competition among ICHCs, as well as to strengthen medical service quality management and improve performance appraisal scheme.

## Introduction

Since the 1990s, with the acceleration of the aging process of the population and the increase in the prevalence of chronic diseases, countries worldwide have put forward the reform direction of building integrated healthcare services [1,2]. Previous studies have reported that integrated care could improve the quality of healthcare [3,4], patient satisfaction [3], and the accessibility of services [5,6]. Since the implementation of the new healthcare system reform in 2009, China has made admirable progress, including the nearly universal insurance coverage, the strengthening of primary healthcare infrastructure, the equalization of basic public health services, the establishment of the national essential medicine system, and the reform in public hospitals [7,8,9,10,11,12,13]. However, China’s healthcare delivery system was fragmented, hospital-centric and treatment-dominated [14,15,16]. Hospitals and primary healthcare centers in China have different functions, operate independently and compete for patients [16]. In 2016, the report *Deepening health reform in China* proposed strengthening health care in China by shaping a tiered healthcare delivery system following a people-centered integrated care model [17,18]. The medical consortium has been vigorously promoted by the National Health Commission as the primary method for achieving people-centered integrated care [19,20]. There are currently four types of medical consortium in China: medical groups in urban areas, integrated county healthcare consortium (ICHC), cross-regional specialist alliances, and telemedicine collaboration networks [19]. The ICHC is composed of a leading county-level hospital, several THCs (CHCs), and village clinics. It is a kind of direct management model in which the leading hospital director is the only legal representative. The leading hospital is responsible for the administrative management and business operation of the personnel, finance, and health resources of the THCs (CHCs) [21,22]. The ICHC has become an important carrier to promote the structural reform of the healthcare supply-side and construct a grading diagnosis and treatment system in rural China. In August 2019, the National Health Commission identified Shanxi Province and Zhejiang Province as pilot provinces for the construction of ICHC, with 567 counties as pilot counties.

The construction of ICHC in Zhejiang Province began in September 2017, when it was decided to carry out pilot work on the construction of ICHC in Deqing County, Dongyang City, Changshan County, Tongxiang City, Keqiao District, Yuyao City, Luqiao District, Chun’an County, Rui’an City, Putuo District, and Jinyun County. 39 county-level hospitals and 170 THCs (CHCs) in the 11 counties (cities and districts) were integrated into 27 ICHCs. By the end of 2019, 70 counties (cities and districts) in Zhejiang formed 208 county-level hospitals and 1,063 THCs (CHCs) into 161 ICHCs, achieving full county coverage of ICHC. The goals of the ICHC are to achieve a community of services, responsibility, interest and management. To achieve these goals, the ICHC in Zhejiang Province has implemented a series of reforms in the management system and operation mechanism. In the management system, almost every county has established an integrated county healthcare consortium management committee, which is generally headed by the county party secretary or governor, with key leaders from the Development and Reform Bureau, the Finance Bureau, the Human Resources and Social Security Bureau, the Health Bureau, and other departments as members. The integrated county healthcare consortium management committee is responsible for major matters such as planning and construction, financial input, personnel arrangement, and assessment and supervision of the ICHC. In the personnel management, medical staffs are recruited, trained, deployed and managed centrally by the ICHC. To encourage doctors from the lead hospital to provide services at the THCs (CHCs), the ICHC usually gives them additional financial subsidies or makes the accumulated service time at the THCs (CHCs) a prerequisite for a title promotion. In terms of financial input, the financial funds allocated by the government to county hospitals and primary healthcare institutions are unified and allocated to the ICHC, which will use them in a coordinated manner, taking into account the nature and purpose of the funds. In terms of health insurance payment methods, the reform of multiple health insurance payment methods under global budget management has been implemented, namely, the inpatient medical service is mainly paid according to the DRGs points method [23], the bed-day payment is gradually implemented for the in-patient medical service of long-term and chronic diseases, and the capitation payment based on the family doctor contract service is implemented for the outpatient medical service [24]. In addition, an incentive and restraint mechanism has been established whereby health insurance savings can be used proportionally by ICHC and reasonable overspending can be shared proportionally between the health insurance administration and ICHC respectively. In order to realize the shift from “treatment-centered” to “health-centered”, Zhejiang Province has made it a key task to strengthen basic public health services and promote the integration of medical and preventive services. The main approach is to select one public health commissioner from the members of the leading group of CDC and one public health liaison from the high-quality professional staffs of CDC, who are then assigned to each to each ICHC simultaneously to supervise and guide public health work. Moreover, public health physicians are encouraged to participate in the family doctor contract services [25]. The prescription for health which was confirmed to be feasible in primary care practice is also regarded as an essential part of clinical medical service [26]. Moreover, the ICHCs in Zhejiang Province have basically realized the unified management of finance, drugs and medical consumables. Zhejiang Province also requires the establishment of medical imaging, electrocardiography, pathology diagnosis and medical testing sharing centers in the county, and ultimately achieve the goal of conducting tests in primary healthcare institutions, county hospitals for diagnosis, and mutual recognition of test results in the county.

The ICHC is a policy-orientated health organization led by the local government [27]. The integration between the leading hospitals and THCs (CHCs) is usually not voluntary, nor is it in response to the healthcare market demands [10]. Previous studies mainly focused on the practice, experience, and short-term performance of ICHC in Zhejiang [28,29,30]. In 2010, Chinese current Premier Li Keqiang pointed out that China should give full play to the role of healthcare professionals as the leading force in the healthcare reform. Inspiring healthcare professionals to get involved plays a vital role in the success of healthcare reform [31,32,33]. However, there are few reports to evaluate this policy based on the empirical data at the healthcare professional level. This study investigated healthcare professionals’ views and satisfaction with this reform and analyzed the problems and improvement measures of this reform.

## Methods

### Data Collection

This cross-sectional study was conducted in the 11 pilot counties (cities and districts) in Zhejiang Province in November 2019. The number of THCs (CHCs) included in the ICHC varies from a minimum of 3 to a maximum of 21, with a mean of 7 and a median of 6. The survey units included the leading hospital of each ICHC, as well as one large, one medium and one small THC (CHC) selected using the convenience sampling method. The questionnaire was developed using an online questionnaire website Wenjuanxing (https://www.wjx.cn/).

The questionnaire link was first sent via WeChat (a Chinese multi-purpose instant messaging, social media app developed by Tencent) to the relevant health bureau administrators in the 11 counties (cities and districts), who then sent the link to the relevant staff in each ICHC office, and finally they sent the link to the DingTalk workgroups (an enterprise communication and collaboration platform developed by Alibaba) of the leading hospital and THCs (CHCs) for online responses by healthcare professionals. The principles of voluntariness, confidentiality, and anonymity were respected throughout the study. The formula of the sample size calculation is N = {Z^2}_{\alpha/2}P(1 - P){\rm{/}}{\delta ^2} [34]. According to a previous study, healthcare professionals’ job satisfaction in the ICHC of Guangxi Autonomous region was 91.15% [35]. Since there was no literature on the healthcare professionals’ satisfaction with ICHC so far, we used this job satisfaction as a reference. The minimum sample size was calculated as 124, giving the study a margin of error of ± 5% (confidence interval 95%).

The sample size in this study was much larger than the minimum sample size calculated.

### Measurement

The self-designed questionnaire consisted of three parts. The first part included sociodemographic information, including the participants’ sex, age, education degree, region, hospital level, job category, professional title, and personnel types. The second part dealt with health Professionals’ views of ICHC, including the healthcare professions’ evaluations of the construction measures, the importance scores of measures to improve the overall medical service capacity, and the severity of the problems of ICHC. The healthcare professions’ perceptions of the construction measures included seven questions, such as the integration of the Centers for Disease Control and Prevention (CDC) and other professional public health institutions into the construction of ICHC can actively promote basic public health work, and so on. Each question was scored using a 5-point response scale where 1 = strongly disagree, 2 = somewhat disagree, 3 = neutral, 4 = somewhat agree, 5 = strongly agree. The importance scores of measures to improve the overall medical service capacity of ICHC involved eight items, such as increasing government financial input and enhancing the security mechanism, and so on. Each item was scored using a 5-point response scale where 5 = very important, 4 = important, 3 = moderately important, 2 = slightly important, 1 = not important. The severity of problems of the ICHC comprised 12 items, such as insufficient financial input, and so on. Each item was scored using a 6-point response scale where 0 meant that the problem did not exist, five meant the problem was the most serious. The higher the score, the stronger the severity of the problem. The third part was the healthcare professional’s satisfaction on the ICHC, which was evaluated with a single item: “Overall, how satisfied are you with the ICHC?” This item was categorized as 0 (not satisfied) and 1 (satisfied).

### Data Analysis

Categorical variables were presented as frequency and percentage. Quantitative variables were described as mean ± standard deviation (SD). Data were also visualized using heatmap, and forest plot. The binary logistic regression analysis with the stepwise forward method was used to investigate the association between sociodemographic variables and satisfaction. The two-tailed *p* < 0.05 was considered to be statistically significant. All analyses were conducted using R 3.6.1 for windows.

## Results

### Demographic Characteristics

A total of 3531 health professionals participated in the study. The socio-demographic characteristics of participants were presented in Table 1. Among the participants, 924 (26.17%) were from Keqiao District, 2563 (72.59%) were from THCs (CHCs), 2229 (63.13%) were female, 2288 (64.8%) aged less than or equal to 40 years old, 2645 (74.91%) had an undergraduate degree, 1911 (54.12%) were clinicians, 1403 (39.73%) were with junior professional title, 3100 (87.79%) were formal staffs.

**Table 1:** Demographic characteristics of participants (N = 3,531).


VARIABLES	CATEGORIES	N (%)

County (City, District)	Deqing County	369 (10.45)

	Dongyang City	213 (6.03)

	Changshan county	237 (6.71)

	Tongxiang City	264 (7.48)

	Keqiao District	924 (26.17)

	Yuyao City	237 (6.71)

	Luqiao District	117 (3.31)

	Chun’an County	207 (5.86)

	Ruian City	345 (9.77)

	Putuo District	306 (8.67)

	Jinyun County	312 (8.84)

Hospital level	Leading county-level hospitals	968 (27.41)

	THCs (CHCs)	2563 (72.59)

Sex	Male	1302 (36.87)

	Female	2229 (63.13)

Age	<=40	2288 (64.80)

	>=41	1243 (35.20)

Education degree	Master or above	60 (1.70)

	Undergraduate degree	2645 (74.91)

	College degree or below	826 (23.39)

Job Category	Clinician	1911 (54.12)

	Public health physician	479 (13.57)

	Medical technician	653 (18.49)

	Administrative personnel	488 (13.82)

Professional title	Senior professional title	573 (16.23)

	Intermediate professional title	1172 (33.19)

	Junior professional title	1403 (39.73)

	No professional title	383 (10.85)

Types of personnel	Formal staff	3100 (87.79)

	Temporary staff	431 (12.21)


### Views of ICHC

**Figure 1:**
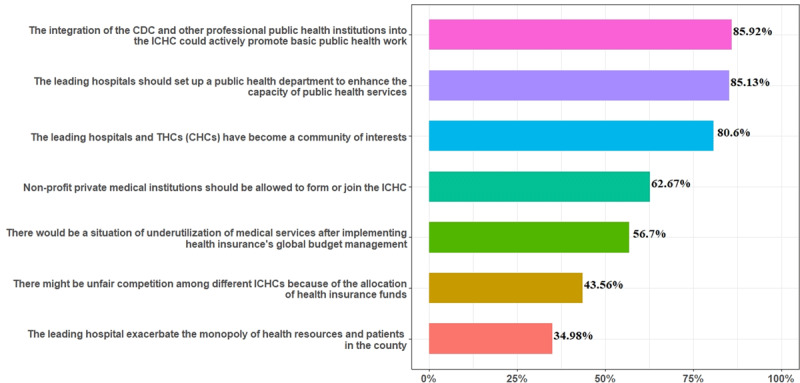
The combined percentage of healthcare professions who responded that they strongly agreed and somewhat agreed with the construction measures of ICHC.

Healthcare professions’ perceptions of the construction measures of ICHC were showed in Figure 1. Among all the participants, 85.92% agreed (including strongly agree and somewhat agree) that the integration of the CDC and other professional public health institutions into the ICHC could actively promote basic public health work. Only 34.98% agreed that the leading hospital has exacerbated the monopoly of health resources and patients in the county.

The severity scores of the problems of the ICHC were presented in Table 2. The most serious problem as perceived by the healthcare professionals was the lack of financial input from the government. The problem with the lowest severity score was poor two-way referral.

**Table 2:** The severity scores of the problems of the ICHC.


PROBLEMS	SCORE (MEAN ± SD)

The government financial fund input is insufficient.	2.92 ± 1.76

There are not enough health professionals.	2.61 ± 1.68

The medical facilities and equipment are poor.	2.48 ± 1.71

The performance appraisal and benefit distribution are unfair.	2.45 ± 1.74

The construction of information sharing platform is lagging.	2.44 ± 1.72

The integration mechanism of medical and preventive services is not perfect.	2.34 ± 1.66

The management system is imperfect.	2.24 ± 1.69

The operational and management autonomy is weakened.	2.23 ± 1.69

The medical service capacity is not strong.	2.17 ± 1.66

The general practitioner contracting services are not as effective as they could be.	1.99 ± 1.72

The payment method of health insurance is unreasonable.	1.92 ± 1.73

The two-way referral system is not smooth.	1.80 ± 1.68


**Figure 2:**
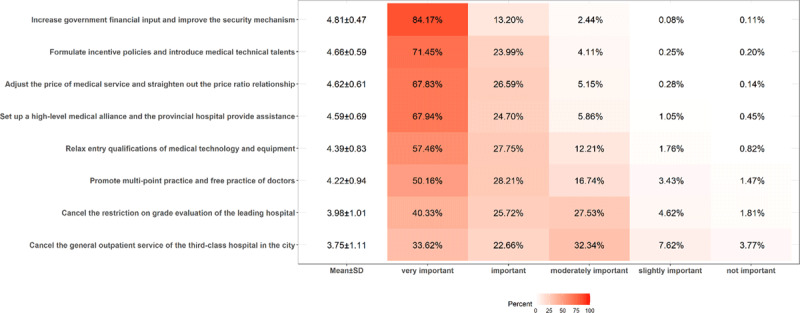
The importance scores of measures to improve the overall medical service capacity of ICHC.

The importance scores of measures to improve the overall medical service capacity of the ICHC were shown in Figure 2. According to the importance scores, from high to low, they were as follows: increase government financial input and improve the security mechanism (4.81 ± 0.47), formulate incentive policies and introduce medical technical talents (4.66 ± 0.59), adjust the price comparison relationship between different medical service items (4.62 ± 0.61), set up a high-level medical alliance and the provincial hospital provides assistance (4.59 ± 0.69), relax access conditions for medical technology and equipment (4.39 ± 0.83), promote multi-location-practice of doctors (4.22 ± 0.94), remove the restriction on the grading evaluation of the leading hospital (3.98 ± 1.01), cancel the general outpatient service of the tertiary hospital in the city (3.75 ± 1.11).

### Satisfaction with ICHC

**Figure 3:**
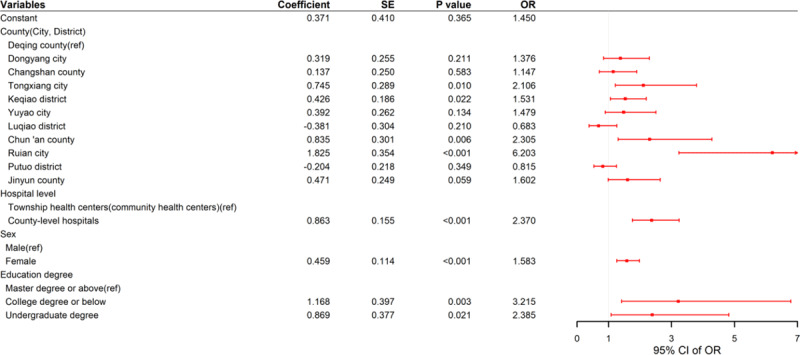
The influencing factors of satisfaction of healthcare professionals to ICHC.

The satisfaction of healthcare professionals to the ICHC was 89.41%. The binary logistic regression model revealed that the county (city, district), hospital level, sex and education degree were associated with satisfaction (Figure 3). It was found that healthcare professionals from the Ruian city, Chun’an county, Tongxiang city, Keqiao district were 6.203 (95% *CI*: 3.235–13.149), 2.305 (95% *CI*: 1.305–4.284), 2.106 (95% *CI*: 1.215–3.793), 1.531 (95% *CI*: 1.059–2.197) times more likely to feel satisfied than healthcare professionals from Deqing county, respectively. The satisfaction was 2.37 (95% *CI*: 1.760–3.238) times more in healthcare professionals from County-level hospitals compared to healthcare professionals from THCs (CHCs). Women’s satisfaction was 1.583 (95% *CI*: 1.265–1.980) times higher than that of men. Healthcare professionals with a college degree or below and an undergraduate degree were 3.215 (95% *CI*: 1.413–6.786) and 2.385 (95% *CI*: 1.084–4.823) times more likely to feel satisfied than those with a master degree or higher, respectively.

## Discussion

With the advancement of ICHC in China, more and more studies have been conducted in this area, but most of them are qualitative studies. In this study, we analyzed healthcare professionals’ views of policies related to the ICHC and overall satisfaction through a cross-sectional questionnaire.

### Integration of Medical Services and Preventive Services

This study showed that nearly 86% of the respondents agreed that the integration of public health institutions into the ICHC can improve the capacity of basic public health services. Surplus of the health insurance fund can be used for employee bonuses, leading hospitals to place more emphasis than before on public health and preventive services to reduce disease incidence and save on medical costs [19]. A recent study revealed that medical staffs were highly satisfied with the integration of medical and preventive services, but there was room for improvement in the interconnection of information systems and data sharing between ICHCs and public health institutions [36].

### Impact of Health Insurance Payment Methods on Health Care Services

Although Zhejiang Province has implemented a variety of different health insurance payment methods for outpatient and inpatient services respectively, 56.7% still agreed that the implementation of global budget management could result in underutilization of medical services. The global budget can effectively curb the growth of insurance fund expenditures and regulate the use of drugs and high-value consumables [37]. However, many studies showed that the implementation of the global budget could not improve quality [37,38]. Furthermore, the cost containment may produce responsibility-shifting behaviors, such as undertreatment of admitted patients, reduction in the number of admissions, prevarication of critically ill patients and those with high medical costs [39].

Similar to the point-of-service (POS) [40], ICHCs allow patients within their jurisdiction to travel outside the county or to other ICHCs for medical treatment. However, the medical expenses incurred by patients are still paid by the ICHC following the health insurance policy. Some researchers are concerned that ICHC may restrict patients’ freedom to seek medical treatment to save the health insurance funds [41], and that ICHCs may induce demand for patients from outside their jurisdictions [42]. However, this study showed that only a small proportion of people agreed that there was unreasonable competition for patients and health insurance funds between different ICHCs, which may be related to some factors such as the implementation of clinical pathways, the creation of health insurance management positions, the implementation of cross-checking and settlement of health insurance funds between ICHCs, and the health professionals tend not to admit restricting patients or inducing demand themselves.

### Government Financial Assistance

Government-subsidized income generally accounts for no more than 10% of the total income of county-level public hospitals [16,43]. The primary healthcare institutions are mainly subsidized by the government [16]. This study indicated that the most serious problem reported by healthcare professionals was inadequate financial input and increasing financial input was considered the most important improvement measure. This may be due to the fact that, on the one hand, some township governments will no longer allocate funds to primary healthcare institutions when they join the ICHC [30], and on the other hand, although county-level hospitals are non-profit hospitals for the public good, they have long been under-invested by the government, and in the face of the uncertainty of the construction of ICHC, county-level hospitals hope to reduce operational risks by increasing financial resources.

### Quantity, Quality and Mobility of Health Professionals

This study revealed that the lack of health professionals was a significant barrier to ICHC. Previous studies also found that lack of primary care practitioners (PCPs), low educational background of PCPs and lack of respect for PCPs were the critical factors affecting the primary medical service ability and gatekeeping role [16,44,45,46]. The China Health Statistics Yearbook 2021 shows that in 2020, the number of licensed (assistant) doctors and registered nurses in primary healthcare institutions accounted for 29.3% and 22.5% of the total, respectively. Among the licensed (assistant) doctors in THCs (CHCs), only 27.2% and 4.0% have bachelor’s degrees and senior titles, respectively. In 2018, the number of general practitioners per 10,000 population in China was 2.22, significantly lower than that of countries such as Canada and Australia [47]. Although ICHCs have issued policies to promote the mobility of healthcare professionals, mainly from county-level hospitals to primary healthcare institutions or from primary healthcare institutions to the same level, there are still many restrictions on the flow of health professionals from primary healthcare institutions to county-level hospitals.

### Establish Urban Medical Alliance with Provincial Hospitals

In China, county-level hospitals mainly provide diagnosis and treatment services for common and frequent-incidence diseases in the county. There is a big gap between county-level hospitals and urban tertiary hospitals in terms of medical service capacity [48]. When county-level hospitals assist primary healthcare institutions, their medical capacity will be weakened. Therefore, in this study, healthcare professionals considered the establishment of a higher level of medical alliance with the help of provincial hospitals as one of the effective improvement measures. In Zhejiang, the cooperation between urban tertiary hospitals and county-level hospitals is a mutually beneficial win-win cooperation model, whereby urban tertiary hospitals send clinical medical experts or managers to county-level hospitals to help them improve their medical ability and management level, and in return, urban tertiary hospitals can not only get financial rewards from the provincial government, but county-level hospitals are usually required to pay a certain percentage (e.g. 3%) of the current year’s revenue to urban tertiary hospitals. One study showed that with the help of urban tertiary hospitals, county-level hospitals improved their management and service capacity and business volume, and also developed new medical technologies. However, county-level hospitals are under tremendous financial pressure [49].

### Performance Appraisal and Benefit Distribution

Although more than 80% of the respondents agreed that the ICHC had become a community of interests, achieving full integration of ICHC remains a challenge if the status of the financial budgets of the healthcare professionals in county-level hospitals and primary healthcare institutions cannot be unified, or if primary healthcare professionals cannot participate fairly in the distribution of benefits. As revealed by this study, the problem of unfair performance evaluation was relatively severe. Other studies reported that there are many problems in the performance appraisal of primary healthcare institutions, such as egalitarianism in public health appraisal, the results of appraisal are not linked to financial subsidies, and the appraisal method does not fully rely on information systems [46,50,51]. In addition, how to promote the equitable distribution of benefits between the staffs of leading hospitals and primary healthcare institutions, between formal and temporary staffs, and between those engaged in medical services and those engaged in public work is one of the important tasks to be addressed by the ICHC.

### Adjustment of Medical Service Prices

Public county-level hospitals and primary healthcare institutions in China have all implemented the zero-markup policy for essential drugs [52]. However, studies showed that the proportion of medical examination and medical consumables income in total medical income increased by substitution, and the proportion of income reflecting the value of medical staffs’ labor and skills remains relatively low [52,53,54]. Therefore, the healthcare professionals in this study agreed that medical service price should be adjusted. Currently, Zhejiang province is actively promoting the dynamic adjustment of medical service prices and gradually increasing the proportion of income from medical technical services by regulating the practice of diagnosis and treatment, strictly controlling the costs of unreasonable medical examinations and laboratory tests.

### Satisfaction and its Influenceing Factors

One study conducted in the Qinghai Province reported that 68.2% of the health professionals in ICHC had job burnout symptoms [55]. Wang et al. [56] revealed that about 14.06% of the primary healthcare professionals in rural China had high turnover intention. A systematic review found that after the healthcare reform in 2009, the job satisfaction of urban community health professionals in China was in the middle level [57]. However, this study showed that health professionals’ satisfaction with ICHC was close to 90%, which may be overestimated to some extent since the county-level health administrators distributed the questionnaire. Besides, we learned in the field interview that primary healthcare providers did not welcome the ICHC so much, especially the directors of THCs, because they were no longer legal representatives and did not have the power of management. As this study showed, the satisfaction of primary healthcare staffs was lower than that of health professionals in county-level hospitals. Some studies reported that primary healthcare providers with higher education are more likely to quit their jobs [58,59], and income was their most dissatisfied aspect [57,60]. In addition, non-financial incentives especially career development opportunities are also important for primary healthcare professionals [61], who still have few opportunities to transfer to the county-level hospitals in the ICHC, which may also contribute to their dissatisfaction. This study showed that health professionals with a master’s degree or higher were less satisfied with the ICHC, which may be related to the fact that highly educated health professionals are more likely to find higher paying jobs in high level hospitals in urban areas.

### Limitations and Suggestions for Further Research

Several limitations should be taken into account when interpreting the results of this study. First, because the county health bureau is the administrative department of the ICHC, the questionnaire link was sent through the administrative staff of the county health bureau, and although it was stated in the questionnaire’s preamble that the survey was anonymous and that the data were for research purposes only, health professionals may still feel some pressure from their superiors to give a positive evaluation. Second, because of the convenience sampling method and online questionnaire used in this study, the health professionals who participated in the survey may have been supporters of ICHC, leading some selection bias and overestimation of satisfaction. Third, the vast majority of the leading hospitals were county-level people’s hospitals, and this study focused on comparing the difference in the views of health professionals in leading hospitals and THCs (CHCs), without analyzing the cases where the leading hospitals were other types of hospitals (e.g., traditional Chinese medicine hospital). Fourth, the purpose of this study was to discover the general attitudes and perceptions of health professionals towards the construction of the ICHC from the perspective of a large sample of data, and in future studies we will use qualitative research methods such as grounded theory, to analyze in depth the reasons behind a specific issue.

## Conclusions

In conclusion, Zhejiang Province has taken adequate measures to promote the construction of ICHC in terms of health insurance payment reform and public health service system. The satisfaction of health professionals with the ICHC was relatively high. At present, the ICHC has not been in operation for a long time, and there are still some problems, such as insufficient financial investment, lack of health professionals and unfair performance appraisal. Further measures are needed in the future to promote the synergy of health insurance, financial, personnel and price policies, to promote competition among different ICHCs, to establish an internal market, to strengthen medical service quality management, and to improve performance appraisal scheme.
